# Research progress on biosynthesis and regulation of monoterpenoid compounds

**DOI:** 10.1007/s13659-026-00640-0

**Published:** 2026-08-24

**Authors:** Junchi Zhang, Jiale Cui, Shang Li, Shiya Chu, Ruiqi Liu, Kangyue Wang, Yuzhe Ren, Changyixin Xiao, Jing Yin

**Affiliations:** 1 Key Laboratory of Saline-Alkali Vegetation Ecology Restoration, Ministry of Education, Harbin, 150040 China; 2 College of AoLin, Northeast Forestry University, Harbin, 150040 China; 3 College of Life Science, Northeast Forestry University, Harbin, 150040 China

**Keywords:** Monoterpenoids, Biosynthesis, Regulation, Metabolic pathways, Biological functions

## Abstract

Monoterpenoids are an important class of plant volatile natural products with broad applications in the food, fragrance, pharmaceutical, and agricultural industries. However, their conventional production largely relies on plant extraction, which is often constrained by low efficiency, high cost, and limited sustainability. Structurally, monoterpenoids can be classified into acyclic, monocyclic, and bicyclic types, and their structural diversity is closely associated with differences in biosynthetic routes and regulatory mechanisms. Their biosynthesis depends on precursor supply from the mevalonate (MVA) and methylerythritol phosphate (MEP) pathways, followed by terpene synthase (TPS)-mediated scaffold formation and subsequent modification reactions. In addition, monoterpenoid accumulation is regulated by multiple factors, including environmental cues, phytohormone signaling, transcriptional regulation, and epigenetic or post-transcriptional control. Meanwhile, substantial progress has been made in the heterologous production of monoterpenoids in microbial platforms such as *Escherichia coli* and *Saccharomyces cerevisiae* through metabolic engineering and synthetic biology. This review summarizes recent advances in monoterpenoid biosynthesis, multilevel regulation, and heterologous production, with particular emphasis on major bottlenecks and optimization strategies for sustainable and efficient biomanufacturing.

**Supplementary Information:**

The online version contains supplementary material available at 10.1007/s13659-026-00640-0.

## Introduction

Monoterpenoids are a diverse class of C10 terpenoid compounds derived from two isoprene units and are widely distributed among plant volatile secondary metabolites. Based on ring systems and functional groups, monoterpenoids are categorized into acyclic, monocyclic, bicyclic, and oxygenated derivatives. As important components of plant volatile organic compounds (VOCs), monoterpenoids not only contribute characteristic aromas but also participate in diverse ecological interactions between plants and their environment [1]. Representative monoterpenoids such as menthol, limonene, and pinene are associated with cooling, fruity, and pine-like odor characteristics, respectively. Beyond their sensory properties, these compounds are closely linked to ecological functions such as pollinator attraction and herbivore deterrence, and they also support broad applications in the flavor and fragrance, essential oil, food, cosmetic, pharmaceutical, and agricultural industries [2, 3]. Due to volatility, structural diversity, bioactivity, and industrial value, monoterpenoids have attracted sustained attention in the fields of natural product chemistry, plant secondary metabolism, and synthetic biology.

However, this approach is often limited by low efficiency, high cost, and strong dependence on plant resources and environmental conditions. These limitations make it difficult to achieve stable and scalable production. In this context, biosynthesis based on metabolic engineering and synthetic biology has emerged as a promising alternative for sustainable monoterpenoid production. Nevertheless, several major challenges remain. First, the biosynthesis of some monoterpenoids is highly complex, involving multistep enzymatic reactions, the generation of multiple isomers, and tight regulation of metabolic branches [4]. Second, the regulatory mechanisms underlying the biosynthesis of several important monoterpenoids are still incompletely understood, particularly for cytochrome P450-mediated downstream modification steps, whose substrate specificity, regioselectivity, and regulatory patterns remain insufficiently characterized [5, 6]. Third, current microbial heterologous production systems still face significant limitations in precursor supply, metabolic flux allocation, product yield, and tolerance to toxic products, all of which hinder industrial-scale biosynthesis.

Therefore, a systematic understanding of monoterpenoid biosynthesis and regulation is essential for clarifying the molecular basis of their structural diversity and for guiding the development of efficient biomanufacturing strategies. This review summarizes the structural characteristics, application value, biosynthesis, and multilevel regulation of representative monoterpenoids, and discusses major bottlenecks and potential optimization strategies for sustainable monoterpenoid production.

## Biological functions and applications of monoterpenoids

**Fig. 1:**
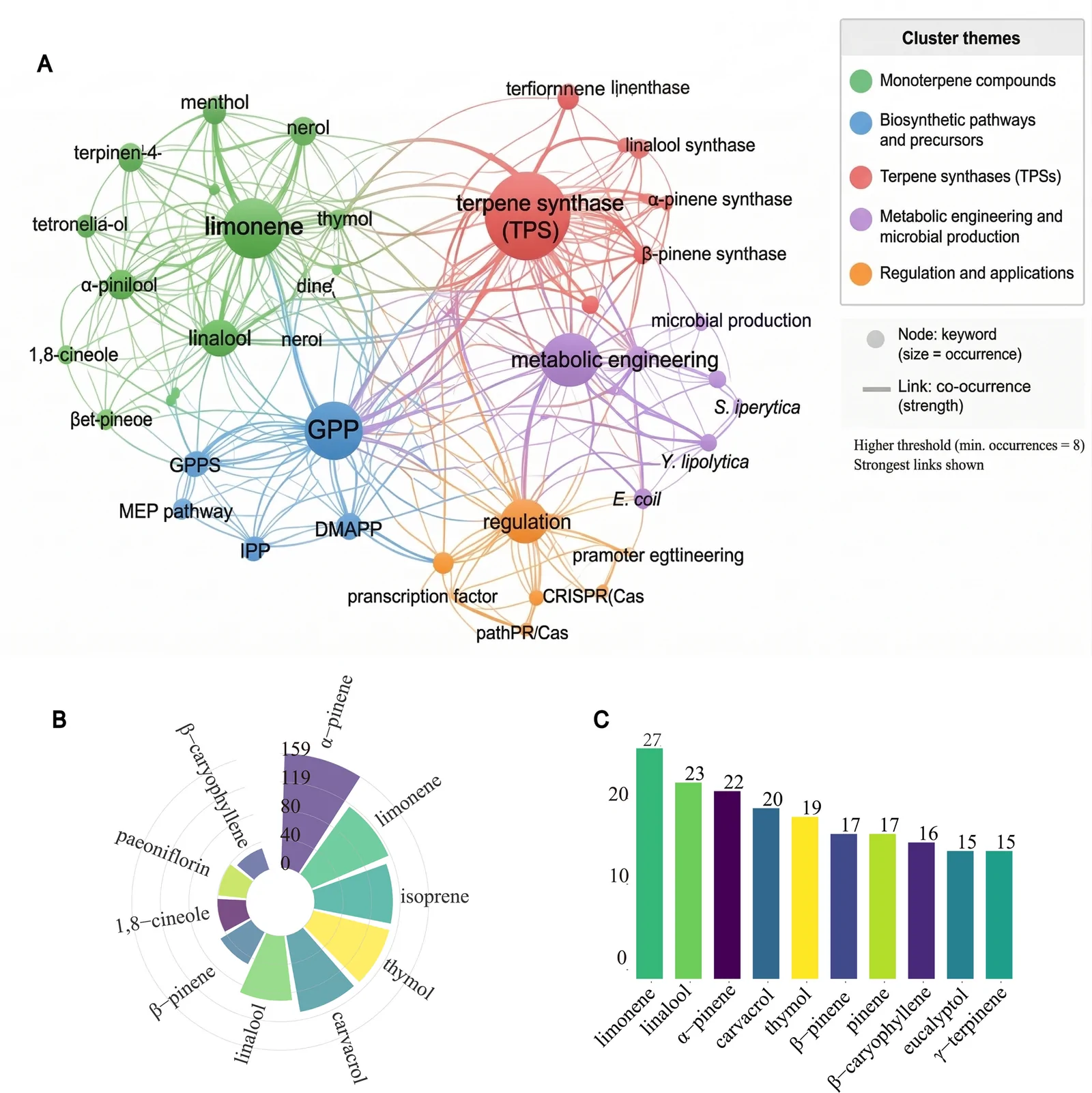
Bibliometric analysis of representative monoterpenoids. **A** Heat map generated by VOSviewer; **B** Circular bar chart showing the frequency of occurrence of the top ten monoterpenoids; **C** Bar chart showing the top ten monoterpenoids based on the number of links

Monoterpenoids are major constituents of plant volatile metabolites and essential oils. They play essential roles in plant–environment communication and adaptive responses. Owing to their volatility, hydrophobicity, and pronounced structural diversity, they participate in a wide range of ecological processes, including pollinator attraction, defense against herbivores, inhibition of pathogenic microorganisms, and mediation of interspecific interactions. In addition, their distinctive odor profiles and diverse bioactivities confer broad application potential in the fragrance and flavor, food, pharmaceutical, and agricultural sectors [1, 7]. In recent years, monoterpenoids have become an important focus in both plant secondary metabolism research and natural product development. Bibliometric analysis of publications indexed in the Web of Science Core Collection over the past five years shows that recent research has mainly focused on representative compounds such as limonene, linalool, α-pinene, carvacrol, thymol, β-pinene, eucalyptol, and γ-terpinene (Fig. 1).

**Fig. 2:**
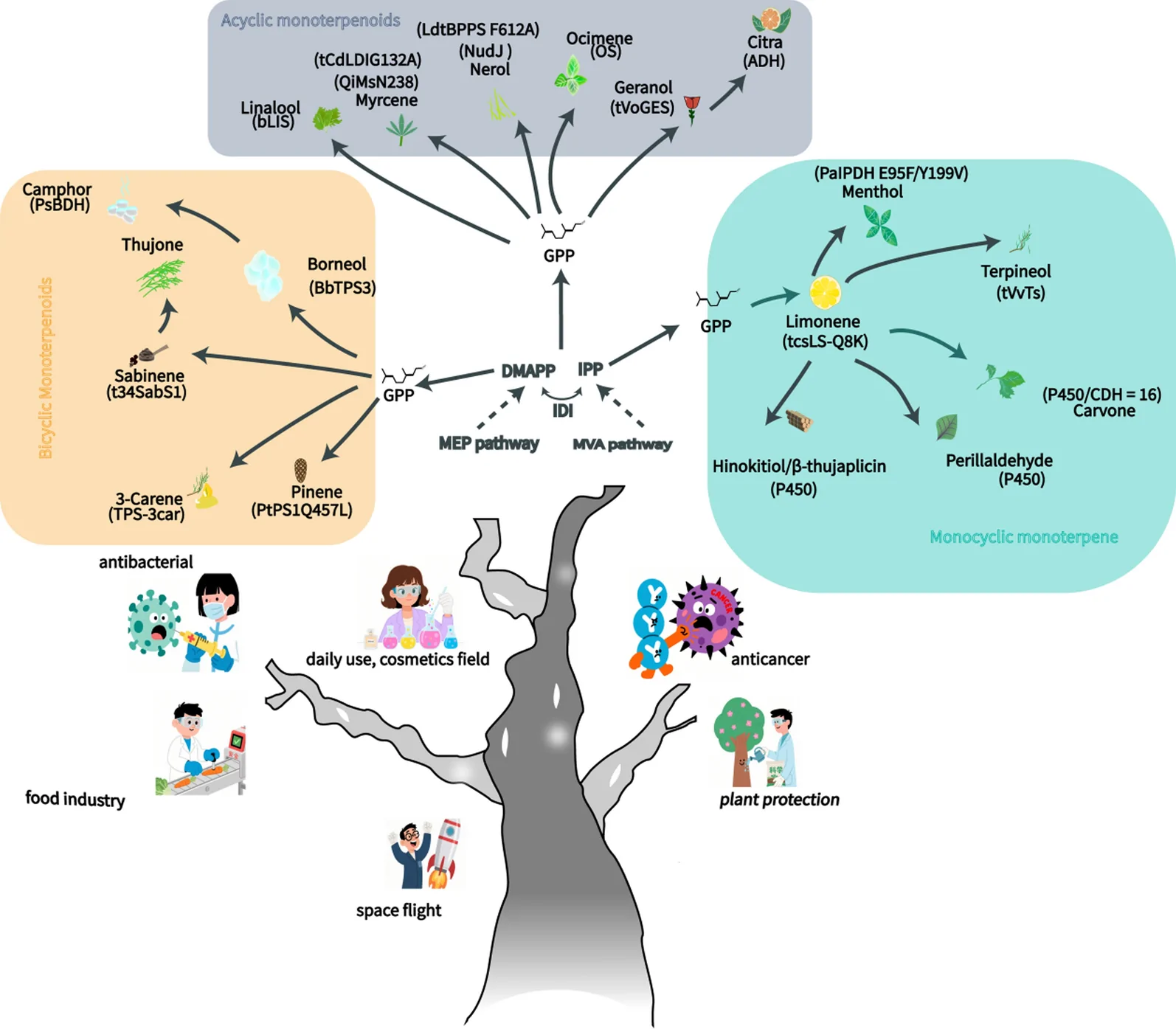
Classification and applications of representative monoterpenoids. IDI, isopentenyl-diphosphate delta isomerase; *tVvTS*, α-terpineol synthase from *Vitis vinifera*; *tcsLS-Q8K*, limonene synthase variant from *Citrus sinensis*; PaIPDH E95F/Y199V, (−)-*(E)*-isopiperitenol dehydrogenase variant from *Pseudomonas aeruginosa*; P450/CDH, P450/(−)-*(E)*-carveol dehydrogenase; *NudJ*, endogenous Nudix hydrolase from *Escherichia coli*; *tVoGES*, geraniol synthase from *Valeriana officinalis*; ADH, alcohol dehydrogenase-type enzyme (geraniol dehydrogenase); *bLIS*, linalool synthase from *Streptomyces clavuligerus*; *QiMsN238*, myrcene synthase from *Quercus ilex*; *tCdLDI* G132A, linalool dehydratase-isomerase variant from *Castellaniella defragrans*; OS, ocimene synthase; *PtPS1Q457L*, pinene synthase variant from *Pinus taeda*; *TPS-3car*, (+)−3-carene synthase; *t34SabS1*, sabinene synthase from *Salvia pomifera*; *BbTPS3*, bornyl diphosphate synthase from *Blumea balsamifera*; *PsBDH*, (+)-borneol dehydrogenase from *Pseudomonas* sp. TCU-HL1. These enzymes represent key catalysts involved in the biosynthesis of the corresponding monoterpenoid products. GPP, geranyl pyrophosphate; IPP, isopentenyl pyrophosphate; DMAPP, dimethylallyl pyrophosphate

From a structural perspective, monoterpenoids can be broadly classified into three groups: acyclic, monocyclic, and bicyclic compounds. These groups differ substantially in odor properties, ecological functions, and application value. Acyclic monoterpenoids, represented by geraniol, nerol, linalool, citral, and myrcene, usually exhibit floral, fruity, or mildly fresh aromas. They are important components of plant volatile blends and play key roles in floral scent formation, pollinator attraction, and other plant–biotic interactions. They also have considerable value in the flavor industry, food aroma formulation, and natural preservation [8–11]. Monocyclic monoterpenoids, such as limonene, menthol, α-terpineol, and carvone, are commonly found in the essential oils of citrus, mint, and perilla. They are typically associated with fresh, citrus-like, minty, or spicy odor characteristics and are therefore widely used in fragrances, food flavoring, oral care products, and personal care products. In addition, some of these compounds exhibit antibacterial, anti-inflammatory, and antioxidant activities [12–20]. Bicyclic monoterpenoids, including α-pinene, borneol, camphor, 3-carene, and thujone, possess more complex ring systems and greater molecular rigidity. Consequently, they often display woody, pine-like, camphoraceous, or medicinal odor notes [21–27]. These compounds have attracted sustained interest in plant defense, pharmacological exploitation, and agricultural applications (Fig. 2).

Overall, the differences among monoterpenoids are not limited to their chemical structures, but also extend to odor properties, ecological roles, and applications, reflecting a clear pattern of structure–function differentiation. Importantly, this differentiation is rooted in differences in biosynthetic logic, including precursor supply, terpene synthase-mediated scaffold formation, and downstream modification reactions.

## Synthesis pathways and regulatory mechanisms of monoterpenoids

### Precursor supply and subcellular origin

Monoterpenoid biosynthesis begins with the supply of universal five-carbon precursors isopentenyl pyrophosphate (IPP) and dimethylallyl pyrophosphate (DMAPP). These precursors are generated through two metabolic pathways located in different subcellular compartments. The MVA pathway operates mainly in the cytosol and uses acetyl-CoA as the starting substrate, and 3-hydroxy-3-methylglutaryl-CoA reductase (HMGR) is an important rate-limiting enzyme. The 2-C-methyl-D-erythritol 4-phosphate (MEP) pathway is localized in the plastids and uses pyruvate and glyceraldehyde-3-phosphate as substrates, and 1-deoxy-D-xylulose-5-phosphate synthase (DXS) is a key regulatory node. The compartmentalized distribution of these two pathways enables plants to regulate precursor supply in a relatively coordinated yet spatially differentiated manner.

**Fig. 3:**
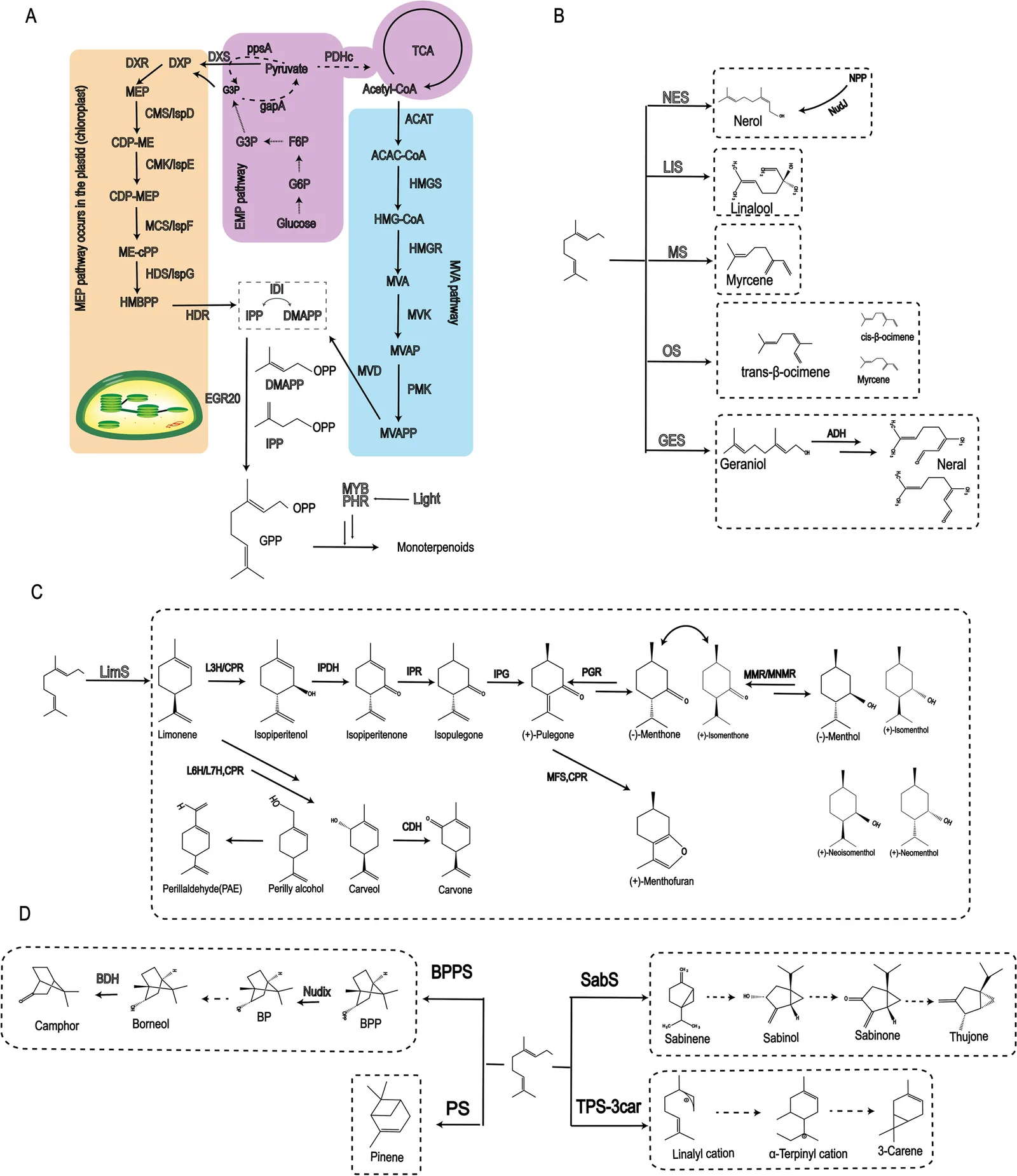
Biosynthetic pathways of representative monoterpenoids. LimS, limonene synthase; L3H, (−)-limonene-3-hydroxylase; CPR, cytochrome P450 reductase; IPDH, (−)-*(E)*-isopiperitenol dehydrogenase; IPR, (−)-isopiperitenone reductase; IPGI, (+)-*(Z)*-isopulegone isomerase; PGR, (+)-pulegone reductase; MMR, (−)-menthone:(−)-menthol reductase; MNMR, menthone:(+)-neomenthol reductase; MFS, (+)-menthofuran synthase; L6H, limonene-6-hydroxylase; L7H, limonene-7-hydroxylase; GES, geraniol synthase; NES, nerol synthase; NudJ, endogenous Nudix hydrolase from *Escherichia coli*; LIS, linalool synthase; MS, myrcene synthase; PS, pinene synthase; BPPS, bornyl diphosphate synthase; BDH, (+)-borneol dehydrogenase; SabS, sabinene synthase. GPP, geranyl pyrophosphate; IPP, isopentenyl pyrophosphate; DMAPP, dimethylallyl pyrophosphate

The IPP and DMAPP produced by the MVA and MEP pathways are subsequently converted by geranyl pyrophosphate synthase (GPPS) into geranyl pyrophosphate (GPP) or its isomer, neryl pyrophosphate (NPP). The precursor supply from the MVA and MEP pathways and the formation of GPP/NPP by GPPS together establish the direct metabolic basis for monoterpenoid biosynthesis (Fig. 3) [28].

### Route diversification from GPP-derived intermediates

Following precursor formation, the structural diversification of monoterpenoids is primarily determined by the different catalytic routes that the common precursor GPP undergoes under the catalysis of terpene synthases (TPSs). Typically, GPP is first ionized to form linalyl diphosphate (LPP) or related carbocation intermediates, which can then give rise to acyclic, monocyclic, or bicyclic skeletons through different reaction modes, including dephosphorylation, cyclization, rearrangement, and termination. These differentiated catalytic pathways form the chemical basis for the structural diversity of monoterpenoids.

Among these routes, 1,6-cyclization is an important reaction mode for forming many monocyclic monoterpenoids and commonly proceeds through an α-terpinyl cation intermediate. This intermediate can generate different products; for example, deprotonation yields limonene, which also serves as an important intermediate for multiple downstream biosynthetic pathways [28]. Subsequent hydroxylation, oxidation, reduction, and isomerization reactions can further produce monocyclic derivatives such as carvone, menthol, and perillaldehyde [29–33].

In contrast, 1,7- or 1,10-cyclization routes generate the rigid frameworks characteristic of bicyclic monoterpenoids and usually involve more complex carbocation rearrangements. For example, pinene synthase catalyzes the cyclization of GPP or NPP to form the bicyclo[3.1.1] heptane skeleton of pinene [34]. Bornyl diphosphate synthase (BPPS) catalyzes the formation of the borneol precursor, which can subsequently be converted into borneol and camphor through hydrolysis and oxidation [24, 35, 36]. Other bicyclic monoterpenoids such as 3-carene and sabinene are generated through related cyclization mechanisms [37].

Compared to cyclic products, the formation of acyclic monoterpenoids retains the open-chain structure of GPP. In this pathway, no cyclization occurs; instead, products are generated through dephosphorylation, water capture, or further oxidation [38]. For example, geraniol and linalool can be formed under the action of specific synthases, while citral is usually produced by further oxidation of geraniol. Acyclic hydrocarbons such as myrcene and ocimene can also be generated through related non-cyclization routes [39–43].

Overall, the formation of acyclic, monocyclic, or bicyclic skeletons from GPP via different catalytic pathways is a key biochemical basis for monoterpenoid diversification and provides the foundation for their subsequent functional differentiation.

### Catalytic motifs and functional diversification of TPSs

During scaffold diversification, the catalytic specificity of TPSs depends not only on their overall folding pattern but also on conserved motifs within the active site that are responsible for metal ion binding and substrate positioning. For Class I TPSs, the DDXXD and NSE/DTE-like motifs are generally regarded as key catalytic elements because they participate in Mg^2^⁺ binding and facilitate substrate ionization, carbocation generation, and subsequent cyclization. Therefore, the composition and spatial organization of these conserved motifs provide an important basis for understanding TPS functional differentiation.

However, increasing evidence indicates that TPS activity does not entirely rely on the strict conservation of classic consensus motifs. A representative example is the geraniol synthase *PgfTPS* from *Penicillium griseofulvum*. Sequence analysis showed that the first metal-binding site contains a DDXXE-type acidic motif variant, and its second metal-binding site is not the typical NSE/DTE-like motif but an atypical NGD motif, where G275 replaces the common serine or threonine residue. Despite these deviations, functional experiments still demonstrated that *PgfTPS* can efficiently catalyze the conversion of GPP to geraniol.

Further structural modeling and molecular docking analyses suggested that PgfTPS still maintained the typical α-helical fold of Class I TPSs and formed an active center conformation compatible with substrate binding and metal ion coordination. Point mutation experiments further revealed that replacing G275 in the NGD motif with serine to simulate a state closer to the classic NSE/DTE-like configuration did not result in a fundamental loss of enzyme activity. These findings indicated that TPS function was not entirely determined by whether a single conserved site exactly matched the consensus sequence. When core motifs vary, other residues around the active pocket may still compensate for metal ion binding, substrate stabilization, or transition state formation through the reconstruction of local interaction networks.

In *PgfTPS*, active pocket residues such as F111, R228, and F352 are believed to contribute to this compensatory effect. Through hydrogen bonding, hydrophobic interactions, and conformational constraints, these residues may collectively stabilize the catalytic center and partially offset the functional risk associated with motif divergence. Thus, the maintenance of TPS catalytic activity relies not only on the classic motifs themselves but also on the cooperative microenvironment of the active center [44].

**Fig. 4:**
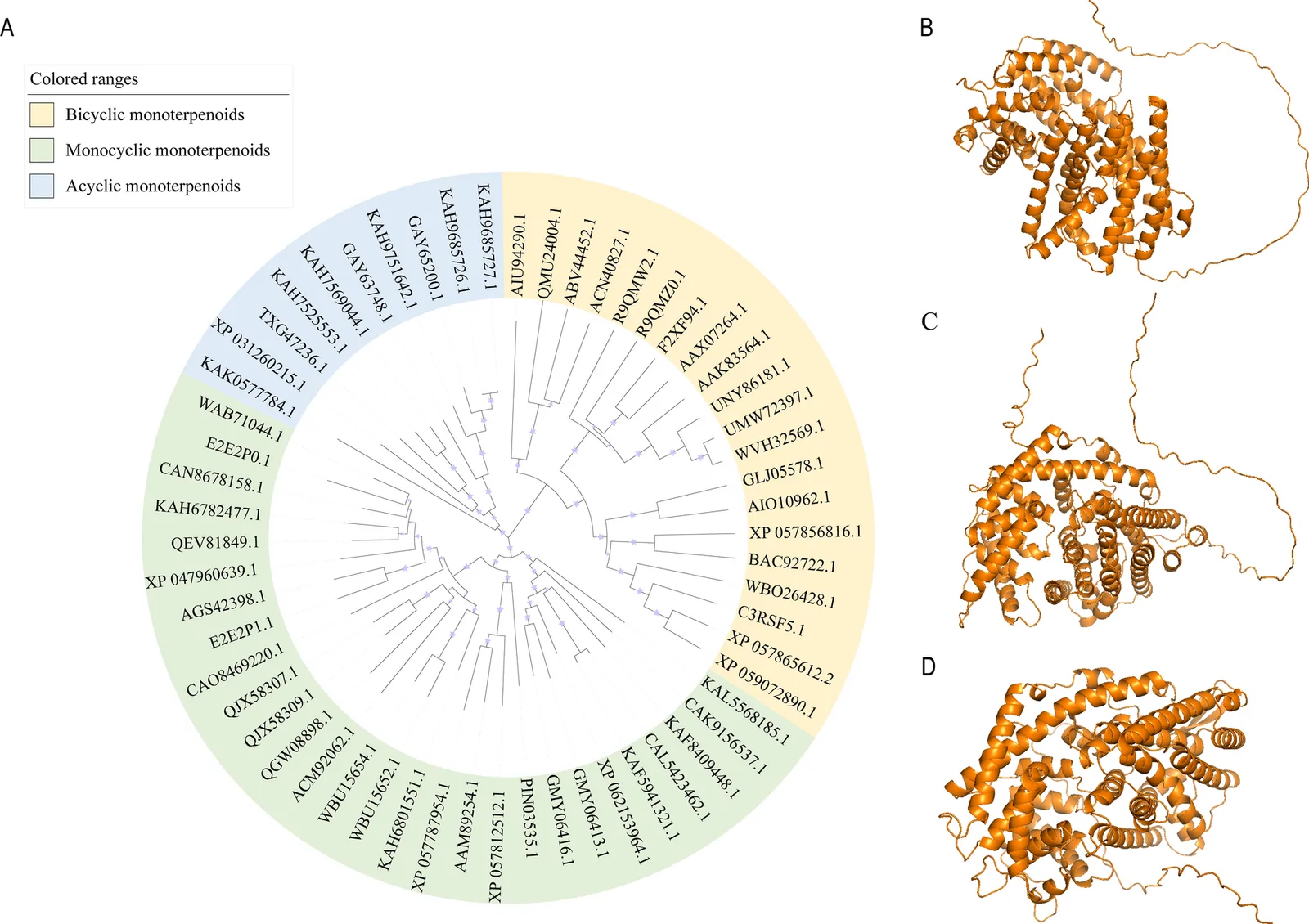
Phylogenetic analysis of representative monoterpenoid synthases and predicted protein structures. **A** Phylogenetic tree of monoterpenoid synthase genes constructed using the Neighbor-Joining (NJ) method in MEGA software with default parameters. The tree is divided into three colored clades based on the type of monoterpenoid products: yellow for bicyclic monoterpenoids, green for monocyclic monoterpenoids, and light blue for acyclic monoterpenoids. **B** Predicted three-dimensional structure of the acyclic monoterpenoid synthase WBU15654A. **C** Predicted three-dimensional structure of the monocyclic monoterpenoid synthase KAH9685727. **D** Predicted three-dimensional structure of the bicyclic monoterpenoid synthase WVH32569

More broadly, this phenomenon of motif variation with retained catalytic function suggests that the TPS diversification may reflect adaptive remodeling of the active center rather than simple changes at individual conserved residues. Furthermore, the TPS evolution often does not occur in isolation. In linear biosynthetic pathways, the skeleton types generated by an upstream TPS constrain the substrate range of downstream tailoring enzymes, especially P450 enzymes [45]. Conversely, changes in the substrate specificity of downstream modifying enzymes may also impose selective pressure on upstream TPS product profiles. Sequence variation in TPSs, structural compensation in the catalytic center, and substrate adaptation of downstream enzymes may therefore evolve in a coordinated manner within the broader biosynthetic system. Finally, the evolutionary relationships among representative acyclic, monocyclic, and bicyclic monoterpenoid synthases are summarized in Fig. 4.

### Multilevel regulation of monoterpenoid biosynthesis

In addition to precursor supply and TPS catalytic specificity, monoterpenoid accumulation is also jointly influenced by a multilevel regulatory network. Overall, environmental signals and endogenous hormones act as upstream inputs, transcription factors function as central hubs linking signal perception to biosynthetic gene expression, and epigenetic or post-transcriptional mechanisms further enhance the regulatory precision and plasticity.

Among environmental factors, light is an important signal influencing monoterpenoid accumulation. In *Mentha canadensis*, low light conditions not only reduce glandular trichome density but also suppress the expression of TPSs and related biosynthetic genes, ultimately decreasing menthol accumulation [46]. These findings indicate that environmental changes can affect monoterpenoid production both by altering the development of specialized structures associated with monoterpenoid synthesis and by directly modulating pathway gene expression. Further studies have shown that light signals can regulate monoterpenoid-related gene expression through transcription factors such as PHR and MYB, highlighting the importance of transcriptional regulatory networks in mediating environmental responses [47].

Endogenous hormone signaling is also an important upstream factor in monoterpenoid regulation, with methyl jasmonate (MeJA) being one of the best-characterized examples. MeJA treatment can significantly induce the expression of monoterpenoid biosynthesis-related genes and is often accompanied by the activation of transcription factor families such as WRKY. In *Mentha canadensis,* MeJA treatment leads to the upregulation of numerous genes, with WRKY family transcription factors showing marked enrichment and a close association with activation of the terpenoid biosynthetic pathway. Together, these observations suggest that both environmental signals and hormonal cues ultimately regulate monoterpenoid biosynthesis through transcription factor-mediated gene expression programs [48].

Beyond transcriptional control, epigenetic and post-transcriptional mechanisms provide additional layers of regulation. In *Mentha canadensis*, methylation differences in promoter regions of key genes such as L3H and L6H indicate that DNA methylation can regulate monoterpenoid synthesis by affecting transcriptional activity. Similarly, in *Schizonepeta tenuifolia*, changes in *FIP37* expression suggest that light intensity may also affect monoterpenoid biosynthesis regulation through m6A RNA methylation-related processes [49]. In addition, miRNA-mediated post- transcriptional regulation contributes to fine-tuning monoterpenoid accumulation. For example, miR156 can regulate *SPL9* and thereby influence *TPS21* expression, forming part of a regulatory cascade that modulates monoterpenoid production [46, 50, 51].

In conclusion, the biosynthesis of monoterpenoids can be viewed as a hierarchical system integrating upstream signal inputs, multi-level gene regulation, and metabolic output. Environmental factors and hormone signals initiate pathway responses, the transcription factor network determines the direction of pathway response, and epigenetic or post-transcriptional mechanisms confer greater precision and adaptability. These interconnected regulatory layers underlie the differential accumulation of monoterpenoids across different species, tissues, and environmental conditions.

## Heterologous biosynthesis of monoterpenoids: progress, bottlenecks, and engineering strategies

### Advantages and research progress of heterologous biosynthesis

**Fig. 5:**
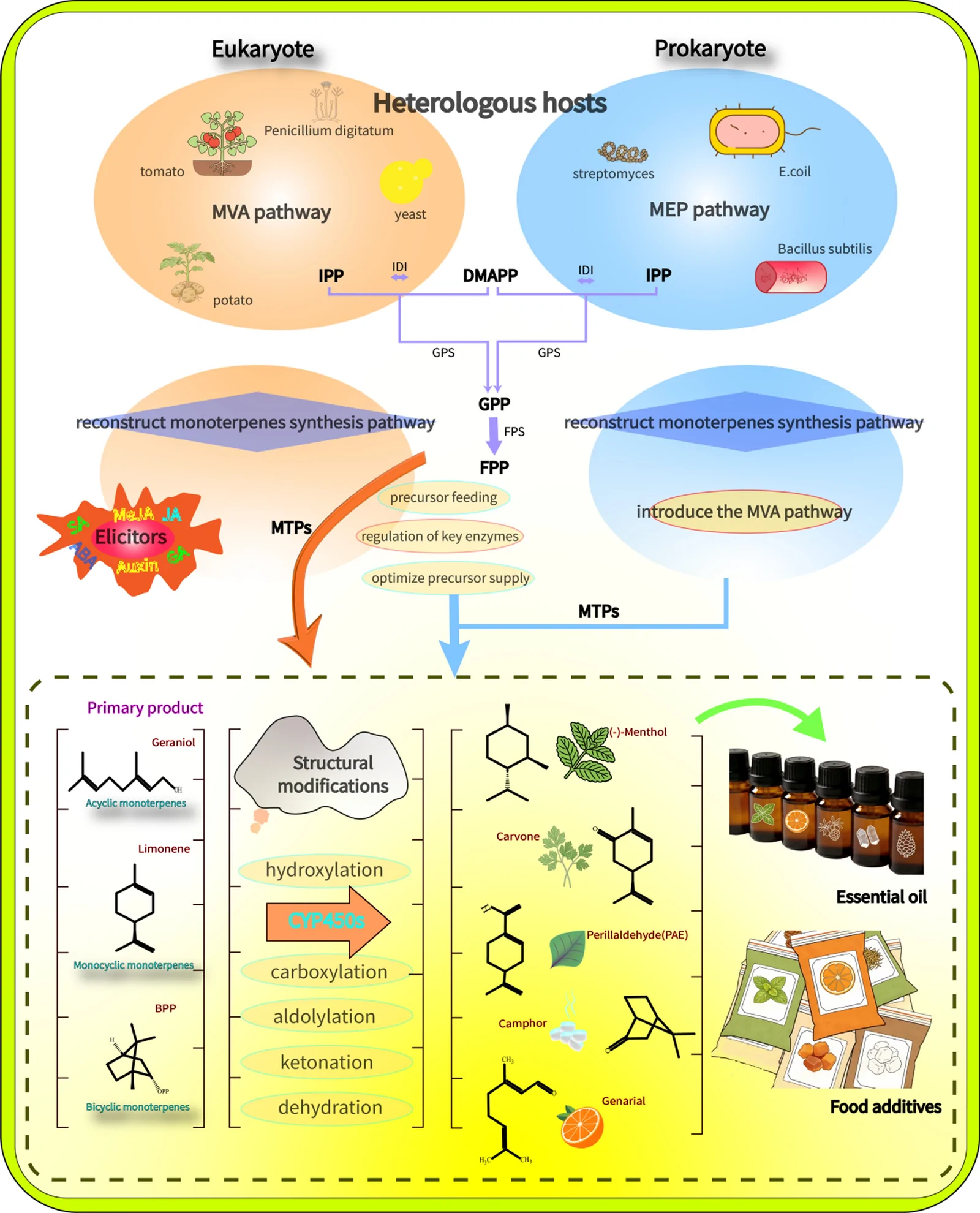
Heterologous biosynthetic strategies for representative monoterpenoids

Traditional approaches for monoterpenoid production mainly rely on plant extraction and chemical synthesis. However, these methods are often limited by high energy consumption, solvent-associated environmental burdens, and poor product specificity, which frequently lead to mixtures of isomers. In contrast, biological synthesis via engineered microbial chassis offers several advantages, such as higher product specificity, the utilization of renewable substrates, and tunability through metabolic engineering. For instance, in yeast and *E. coli* systems, the ability to optimize precursor pathways and enzyme functions has enabled the targeted production of specific monoterpenoid products while reducing by-products and improving overall efficiency [27, 52]. These advances highlight the potential of microbial biosynthesis as a promising platform for the sustainable and scalable production of monoterpenoids (Fig. 5) [53].

Current heterologous monoterpenoid production strategies can be broadly classified into three categories: whole-cell microbial factories, cell-free enzymatic systems, and microbial biotransformation platforms. These systems differ in precursor utilization, enzyme performance, product tolerance, and process control, and together they constitute the main technological routes for producing representative monoterpenoids such as limonene, menthol, carvone, geraniol, linalool, myrcene, pinene, borneol, camphor, and sabinene. To provide a concise overview of the current landscape of heterologous monoterpenoid production, the representative production platforms, major engineering strategies, and best-performing cases for the principal monoterpenoids discussed in this review are summarized in Table 1. To avoid excessive complexity in the main text while retaining dataset completeness, the detailed records for whole-cell microbial production, cell-free synthesis, and microbial biotransformation are provided in Tables S1–S3, respectively.

**Table 1:** Major engineering strategies and best-performing cases

MTP	Heterologous host	Key detailed engineering strategy	Titer		References
Limonene	*E. coli*	Directed evolution of *Citrus sinensis* limonene synthase; MVA pathway enhancement; fermentation process optimization	4.9 g/L	[54]	
Limonene	*S. cerevisiae*	Downregulation of ERG20; mitochondrial compartment engineering; precursor and cofactor supply enhancement; fermentation optimization	2.63 g/L	[55]	
Geraniol	*Candida glycerinogenes*	Dynamic regulation of competitive pathways; expansion of substrate utilization; energy metabolism balancing	5.2 g/L	[56]	
Geraniol	*E. coli*	Fusion tag engineering for enzyme stability; MVA pathway overexpression; boosting of GPP precursor pool	4.8 g/L	[57]	
Linalool	*S. cerevisiae*	Directed evolution of linalool synthase; complete MVA pathway overexpression; redox balance optimization	5.87 g/L	[58]	
α-Terpineol	*Penicillium digitatum*	Biotransformation condition optimization using response surface methodology (RSM)	5.87 g/L	[59]	
Carvone	*E. coli*	Balanced expression of P450 limonene-6-hydroxylase and carveol dehydrogenase (CDH)	4.3 g/L	[60]	
(+)-α-Pinene	*E. coli*	Genetic element optimization; pinene synthase engineering; MEP pathway strengthening; oxidation module assembly	1.87 g/L	[61]	
(−)-Menthol	*E. coli*	Discovery and engineering of bacterial (+) -pulegone reductase; MEP pathway tuning; one-pot biocatalysis	1.5 g/L	[62]	

### Universal bottlenecks and type-specific challenges

Despite rapid progress in heterologous biosynthesis, large-scale production of monoterpenoids still faces several common bottlenecks. First, insufficient supply of GPP remains a major limitation. In many microbial hosts, endogenous metabolic pathways compete for precursor pools; for example, sterol biosynthesis in yeast diverts carbon flux away from monoterpenoid formation. As a result, even after the introduction of heterologous biosynthetic modules, target pathways often remain constrained by limited precursor availability. Second, heterologously expressed TPSs and downstream modifying enzymes frequently show low catalytic efficiency, poor substrate specificity, or unstable expression, all of which directly affect product yield and selectivity. Third, many monoterpenoids and their intermediates are highly volatile, membrane-disruptive, or metabolically stressful, thereby inhibiting host growth and reducing fermentation stability. In addition to these general constraints, different classes of monoterpenoids face distinct challenges. For acyclic monoterpenoids, open-chain intermediates are more susceptible to degradation or diversion by endogenous phosphatases, oxidases, and other non-specific enzymes, making intermediate stability a key concern. Monocyclic monoterpenoids often require longer downstream modification cascades, including hydroxylation, reduction, and isomerization, which increases the difficulty of pathway balancing and coordinated enzyme expression. For bicyclic monoterpenoids, product formation relies more strongly on precise TPS catalysis and complex carbocation rearrangements, and is therefore often limited by low cyclization efficiency and insufficient product selectivity. Taken together, these observations indicate that monoterpenoid production cannot be achieved by a single universal solution, but instead requires bottleneck-oriented engineering tailored to individual product types.

### Core optimization strategies

To address these bottlenecks, several broadly applicable engineering strategies have been developed. Among them, enhancement of precursor supply is a fundamental approach for increasing monoterpenoid yield. Common strategies include reinforcing rate-limiting steps in the MVA or MEP pathways, such as overexpression of tHMG1 or DXS, or introducing heterologous precursor utilization routes such as the isopentenol utilization pathway (IUP), thereby increasing carbon flux toward GPP formation. Enzyme engineering is another key strategy for improving catalytic efficiency and product specificity. Through directed evolution, site-directed mutagenesis, or fusion expression with auxiliary proteins, TPSs and downstream modifying enzymes can be optimized for better substrate recognition, higher turnover rates, and improved product selectivity. For monoterpenoids that require multistep downstream conversion, enzyme engineering is often combined with coordinated optimization of the entire pathway module.

Subcellular compartmentalization and dynamic regulation also provide effective solutions to precursor competition, intermediate loss, and product toxicity. Targeting key biosynthetic modules to organelles such as mitochondria or peroxisomes can partially isolate the pathway from endogenous competing reactions while reducing intermediate degradation and product-associated toxicity. In parallel, promoter-mediated control of competing pathways, fed-batch fermentation, and two-phase culture systems can help maintain metabolic balance and improve process robustness. For some structurally complex monoterpenoids, cell-free synthetic biology offers an additional option by bypassing cellular growth constraints and directly constructing modular in vitro enzyme systems. Overall, efficient heterologous production of monoterpenoids usually depends on the combined implementation of multiple strategies rather than on any single intervention alone (Table 1).

### Representative case studies and strategic insights

Representative case studies further illustrate how engineering strategies can be matched to product-specific bottlenecks. Limonene provides a typical example in which precursor pathway enhancement and enzyme engineering act synergistically. Because limonene biosynthesis is strongly constrained by insufficient GPP supply, researchers reconstructed the MVA pathway in *E. coli* to improve carbon flux toward GPP accumulation. At the same time, limonene synthase was optimized to enhance substrate utilization and reduce by-product formation. In one representative study, targeted engineering of citrus-derived limonene synthase, together with pathway optimization, enabled limonene production to reach 4.90 g/L in *E. coli* [54]. This case demonstrates that precursor enhancement combined with key enzyme optimization is particularly effective for addressing flux limitation and catalytic inefficiency in monocyclic monoterpenoid production.

Myrcene, by contrast, highlights the value of compartmentalization strategies for acyclic monoterpenoid production. Because acyclic monoterpenoids are more vulnerable to open-chain intermediate degradation and product toxicity, precursor enhancement alone is often insufficient to achieve stable high-level production. In this case, key biosynthetic modules were targeted to mitochondria, which not only reduced non-specific consumption of intermediates in the cytosol but also alleviated the toxic effects of myrcene on the host. When combined with fed-batch fermentation and related process optimization, this strategy enabled a marked increase in myrcene production in *S. cerevisiae*, reaching 142.64 mg/L [63]. This example indicates that, for acyclic monoterpenoids, subcellular compartmentalization can simultaneously protect unstable intermediates, reduce product toxicity, and improve overall pathway efficiency. Taken together, the cases of limonene and myrcene illustrate two representative optimization logics in heterologous monoterpenoid biosynthesis. Precursor enhancement combined with enzyme engineering is particularly effective for addressing flux limitation and catalytic inefficiency in monocyclic monoterpenoids, whereas compartmentalization coupled with process optimization is more suitable for mitigating intermediate degradation and product toxicity. Future development of monoterpenoid biomanufacturing should therefore move beyond generic parameter optimization and place greater emphasis on product type, bottleneck identification, and mechanism-guided strategy integration. Only by establishing a rational framework of bottleneck identification, strategy matching, and system-level coordination can monoterpenoid production be further advanced toward efficient, stable, and industrially feasible green manufacturing.

## Conclusion and perspectives

Monoterpenoids are a structurally diverse class of natural products with important ecological functions and broad application potential in the food, fragrance, pharmaceutical, agricultural, and related industrial sectors. Owing to their bioactivities and commercial value, they have attracted growing attention in natural product chemistry, plant secondary metabolism, and synthetic biology. However, the production of many high-value monoterpenoids still depends largely on plant extraction, which is often constrained by seasonal variation, climatic conditions, unstable raw material supply, and limited sustainability. These limitations underscore the need for efficient and controllable biosynthetic routes.

Substantial progress has been made in recent years in elucidating monoterpenoid biosynthesis, including precursor supply through the MVA and MEP pathways, TPS-mediated scaffold diversification, and downstream modification reactions that contribute to product complexity. At the regulatory level, increasing evidence indicates that monoterpenoid accumulation is governed by multilevel control involving environmental cues, phytohormone signaling, transcription factors, and epigenetic or post-transcriptional mechanisms. In parallel, heterologous production platforms based on microbial cell factories, cell-free systems, and biotransformation strategies have considerably expanded the toolbox for monoterpenoid production. Nevertheless, progress remains uneven across different compounds. While representative products such as limonene, menthol, and geraniol have achieved notable advances [64], investigation into thujone and other compounds has only recently begun.

Future research should therefore place greater emphasis on integrating mechanistic understanding with engineering innovation. Key priorities include improving enzyme specificity and catalytic efficiency, strengthening precursor supply, enhancing host tolerance to toxic intermediates and products, and optimizing pathway coordination for complex downstream modification steps. In this context, AI-assisted protein engineering, chassis optimization, modular pathway design, and cell-free or omics-guided approaches are likely to play increasingly important roles [65, 66]. With continued advances in synthetic biology, metabolic engineering, and enzyme design, a broader range of monoterpenoids is expected to become accessible through sustainable and scalable biomanufacturing. Such progress will not only deepen our understanding of monoterpenoid diversity, but also accelerate their practical application in green production systems.

## Supplementary Information

Additional file1 (PDF 298 KB)

## Data Availability

No new datasets were generated or analyzed during the current study. All data discussed in this review are derived from previously published studies and are available from the cited references.
