# Supplementary material for: Research progress on biosynthesis and regulation of monoterpenoid compounds
Source: Nat Prod Bioprospect. 2026 Aug 24;16(1):82. doi: 10.1007/s13659-026-00640-0 (PMC13504028; doi:10.1007/s13659-026-00640-0)
Supplement: Supplementary file 1 [file 13659_2026_640_MOESM1_ESM.pdf]

Research Progress on Biosynthesis and Regulation of Monoterpenoid Compounds

Junchi Zhang [a,b#], Jiale Cui [c#], Shang Li [c], Shiya Chu [b], Ruiqi Liu [b], Kangyue Wang [b], Yuzhe Ren [c], Changyixin Xiao [c], Jing Yin [a,c] *

a. Key Laboratory of Saline-alkali Vegetation Ecology Restoration, Ministry of Education, Harbin, 150040, China

b. College of AoLin, Northeast Forestry University, Harbin, 150040, China

c. College of Life Science, Northeast Forestry University, Harbin, 150040, China

# Junchi Zhang and Jiale Cui are co-first authors, and two authors contributed equally.

Table S1: Whole-cell microbial production of representative monoterpenoids

MTP	Heterologous host	Key Engineering Strategy	Output (g/L)	Reference
Limonene	*Saccharomyces cerevisiae*	OLB pathway construction; Glucose-sensing promoter downregulating ERG20; MVA pathway enhancement; Limonene synthase engineering	0.9177	[1]
Limonene	*S. cerevisiae*	Downregulating ERG20; Enzyme optimization; Mitochondrial engineering; Fermentation process optimization; Enhancement of precursor and cofactor supply	2.63	[2]
Limonene	*Escherichia coli*	Enzyme engineering; MVA pathway; Fermentation process optimization	4.9	[3]
Menthol	*S. cerevisiae*	MVA pathway enhancement; Dynamic regulation of competitive pathways; Downstream pathway balance optimization	0.0063	[4]

MTP	Heterologous host	Key Engineering Strategy	Output (g/L)	Reference
Menthol	*E. coli*	MEP pathway optimization; Enzyme engineering	/	[5]
Menthol	*E. coli*	Enzyme discovery & engineering; MEP pathway; One-Pot Biocatalysis	1.5	[6]
α-Terpineol	*Penicillium digitatum*	Response Surface Methodology (RSM)	0.8339	[7]
α-Terpineol	*Sphingobium* sp.	Biotransformation condition optimization	240	[8]
α-Terpineol	*S. cerevisiae*	Enzyme Engineering; MVA pathway; Competitive pathway rewiring	0.0219	[9]
Carvone	*E. coli*	Balanced expression of P450/CDH16; Heterologous expression of plant-derived CYP71D18-ATR2-CD pathway	0.044	[10]
Perillaldehyde (PAE)	*E. coli*	MVA pathway; Enzyme engineering; Fed-Batch fermentation	0.415	[11]
Geraniol	*E. coli*	Fusion Tag engineering; MVA pathway	4.8	[12]
Geraniol	*S. cerevisiae*	Enzyme engineering; MVA pathway; Precursor (GPP) synthesis regulation	1.4	[13]
Geraniol	*Candida glycerinogenes*	Dynamic Regulation of Competitive Pathway; Expansion of Substrate Utilization; Bioprocess Optimization	5.2	[14]
Nerol	*E. coli*	Novel precursor pathway; MVA pathway	0.415	[15]
Nerol	*E. coli*	Novel Precursor Pathway; Site-Directed mutagenesis; Dephosphorylation enhancement	0.76	[16]
Linalool	*E. coli*	Bioinformatics-aided Enzyme Discovery	/	[17]
Linalool	*E. coli*	MEP pathway; Fermentation process optimization	1.52	[18]

MTP	Heterologous host	Key Engineering Strategy	Output (g/L)	Reference
Linalool	*S. cerevisiae*	MVA pathway; Enzyme engineering	5.87	[19]
Linalool	*S. cerevisiae*	IUP pathway; Enzyme engineering	5.2	[20]
Myrcene	*E. coli*	Enzyme Engineering; MVA pathway; Subcellular compartmentalization	0.0582	[21]
Myrcene	*S. cerevisiae*	Enzyme engineering; Host metabolic engineering; Advanced bioprocess design	0.0668	[22]
Myrcene	*S. cerevisiae*	Enzyme engineering; MVA pathway; Subcellular Compartmentalization	0.1426	[22]
Myrcene	*E. coli*	Enzyme engineering; High-Yield Geraniol Platform	5.73	[23]
α/β-Pinene	*E. coli*	Enzyme engineering; MVA pathway	/	[24]
α/β-Pinene	*E. coli*	Genetic element optimization; Enzyme Engineering; Precursor Pathway Optimization; Oxidation Module	1.87	[25]
α/β-Pinene	*E. coli*	Enzyme engineering; MVA pathway	1.0351	[26]
α/β-Pinene	*Rhodobacter sphaeroides*	MEP pathway; Genetic expression optimization	0.0001488	[27]
α/β-Pinene	*Yarrowia lipolytica*	MVA Pathway; Low-Cost Feedstock Utilization	0.5056	[28]
Borneol	*S. cerevisiae*	Enzyme engineering & discovery; MVA pathway	/	[29]
Borneol	*S. cerevisiae*	MVA pathway; Enzyme discovery & characterization	/	[30]
Borneol	*S. cerevisiae*	Enzyme engineering	0.1486	[31]

MTP	Heterologous host	Key Engineering Strategy	Output (g/L)	Reference
Borneol	*S. cerevisiae*	MVA pathway; Phosphatase discovery and engineering; Balancing cellular dephosphorylation metabolism	/	[32]
Borneol	*S. cerevisiae*	Enzyme engineering; MVA pathway	0.0230	[33]
Borneol	*E. coli*	Orthogonal precursor pathway; Enzyme engineering; Precursor Dephosphorylation Enhancement	0.0872	[16]
Camphor	*S. cerevisiae*	Enzyme engineering; MVA pathway	0.0235	[33]
Sabinene	/	Enzyme discovery	/	[34]
Sabinene	*E. coli*	Adaptive laboratory evolution; Multi-Omics analysis; Reverse engineering for validation	/	[35]
Sabinene	*S. cerevisiae*	Subcellular compartmentalization; Enzyme engineering & fusion; MVA pathway	0.1549	[36]

Table S2 Cell-free synthesis of representative monoterpenoid

MTP	Reaction system	Key Engineering Strategy	Output (g/L)	Reference
Limonene	Cell-Free Protein Synthesis (CFPS)	In vitro pathway reconstitution	0.610	[37]
Myrcene	Hybrid cell-free reaction system	Multi-enzyme cascade module assembly	/	[38]
Sabinene	Cell-free system	Computational modeling for bottleneck identification; ATP regeneration and phosphate cycling; Cofactor recycling and redox balancing	15.9	[39]

Table S3 Microbial biotransformation routes for representative monoterpenoids

MTP	Microbial strain	Transformation Strategy	Output (g/L)	Reference
Thujone	*Absidia coerulea*	Fungal whole-cell biotransformation	/	[40]

Reference:

[1] Cheng S, et al. Orthogonal Engineering of Biosynthetic Pathway for Efficient Production of Limonene in Saccharomyces cerevisiae. ACS Synth Biol, 2019, 8(5): 968–975. https://doi.org/10.1021/acssynbio.8b00544

[2] Kong X, et al. Efficient Synthesis of Limonene in Saccharomyces cerevisiae Using Combinatorial Metabolic Engineering Strategies. J Agric Food Chem, 2023, 71(20): 7752–7764. https://doi.org/10.1021/acs.jafc.3c00886

[3] Scipion CP, et al. Enhanced Production of (+)-Limonene through Targeted Engineering of Citrus sinensis Limonene Synthase. ACS Synthetic Biology, 2025. https://doi.org/10.1021/acssynbio.4c00645

[4] Lv X, et al. Engineered Saccharomyces cerevisiae for the De Novo Biosynthesis of (-)-Menthol. Journal of Fungi, 2022, 8(9): 982. https://doi.org/10.3390/jof8090982

[5] Zhan J, et al. Discovery and engineering of bacterial (−)-isopiperitenol dehydrogenases to enhance (−)-menthol precursor biosynthesis. Advanced Synthesis & Catalysis, 2021, 363(16): 3973–3982. https://doi.org/10.1002/adsc.202100476

[6] Wu X, et al. Biosynthesis of pinene in purple non-sulfur photosynthetic bacteria. Microbial Cell Factories, 2021, 20(1): 104. https://doi.org/10.1186/s12934-021-01556-2

[7] Tai YN, et al. Optimisation of alpha-terpineol production by limonene biotransformation using Penicillium digitatum DSM 62840. J Sci Food Agric, 2016, 96(3): 954–961. https://doi.org/10.1002/jsfa.7302

[8] Molina G, et al. Optimization of limonene biotransformation for the production of bulk amounts of alpha-terpineol. Bioresour Technol, 2019, 294: 122180. https://doi.org/10.1016/j.biortech.2019.122180

[9] Zhang C, et al. Alpha-Terpineol production from an engineered Saccharomyces cerevisiae cell factory. Microbial Cell Factories, 2019, 18(1): 160. https://doi.org/10.1186/s12934-019-1127-1

[10] Yoshida E, et al. Increased carvone production in Escherichia coli by balancing limonene conversion enzyme expression via targeted quantification concatamer proteome analysis. Scientific Reports, 2021, 11(1): 22126. https://doi.org/10.1038/s41598-020-79596-9

[11] Sun C, Dong X, Zhang R. Effectiveness of recombinant Escherichia coli on the production of (R)-(+)-perillyl alcohol. BMC Biotechnology, 2021, 21: 3. https://doi.org/10.1186/s12896-020-00668-7

[12] Wang X, et al. Engineering Escherichia coli for production of geraniol by systematic synthetic biology approaches and laboratory-evolved fusion tags. Metab Eng, 2021, 66: 60–67. https://doi.org/10.1016/j.ymben.2021.03.009

[13] Zhao J, et al. Improving monoterpene geraniol production through geranyl diphosphate synthesis regulation in Saccharomyces cerevisiae. Applied Microbiology and Biotechnology, 2016, 100(10): 4561–4571. https://doi.org/10.1007/s00253-016-7309-4

[14] Zhao C, et al. Metabolic Engineering of Candida glycerinogenes for Sustainable Production of Geraniol. ACS Synth Biol, 2023, 12(6): 1836–1844. https://doi.org/10.1021/acssynbio.3c00084
[15] Zong Z, et al. Biosynthesis of nerol from glucose in the metabolically engineered Escherichia coli. Bioresource Technology, 2019. https://doi.org/10.1016/j.biortech.2019.121654
[16] Lei D, et al. Combining Metabolic and Monoterpene Synthase Engineering for de Novo Production of Monoterpene Alcohols in Escherichia coli. ACS Synth Biol, 2021, 10(6): 1531–1544. https://doi.org/10.1021/acssynbio.0c00584
[17] Zhang C, et al. Bioinformatics-aided identification, characterization and applications of mushroom linalool synthases. Communications Biology, 2021, 4(1): 223. https://doi.org/10.1038/s42003-020-01568-7
[18] Wang X, et al. Efficient biosynthesis of R-(−)-linalool through adjusting the expression strategy and increasing GPP supply in Escherichia coli. Journal of Agricultural and Food Chemistry, 2020, 68(31): 8381–8390. https://doi.org/10.1021/acs.jafc.0c04014
[19] Zhou P, et al. Improved linalool production in Saccharomyces cerevisiae by combining directed evolution of linalool synthase and overexpression of the complete mevalonate pathway. Biochemical Engineering Journal, 2020, 161: 107655. https://doi.org/10.1016/j.bej.2020.107655
[20] Zhang Y, et al. Enhancement of linalool production in Saccharomyces cerevisiae by utilizing isopentenol utilization pathway. Microbial Cell Factories, 2022, 21(1): 212. https://doi.org/10.1186/s12934-022-01716-7
[21] Kim EM, et al. Microbial Synthesis of Myrcene by Metabolically Engineered Escherichia coli. J Agric Food Chem, 2015, 63(18): 4606–4612. https://doi.org/10.1021/acs.jafc.5b02217
[22] Shu Y, et al. Systematic Engineering to Enhance beta-Myrcene Production in Yeast. J Agric Food Chem, 2024, 72(35): 19395–19402. https://doi.org/10.1021/acs.jafc.4c02909
[23] Wang X, et al. Efficient myrcene production using linalool dehydratase isomerase and rational biochemical process in Escherichia coli. J Biotechnol, 2023, 371-372: 33–40. https://doi.org/10.1016/j.jbiotec.2023.05.004
[24] Tashiro M, et al. Bacterial Production of Pinene by a Laboratory-Evolved Pinene-Synthase. ACS Synth Biol, 2016, 5(9): 1011–1020. https://doi.org/10.1021/acssynbio.6b00168
[25] Zhou Y, et al. Efficient Biosynthesis of (+)-α-Pinene and de Novo Synthesis of (+)-cis-Verbenol in Escherichia coli. Journal of Agricultural and Food Chemistry, 2024, 72(34): 18890–18897. https://doi.org/10.1021/acs.jafc.4c02645
[26] Zhou Y, et al. Efficient production of α-pinene through identifying the rate-limiting enzymes and tailoring inactive terminal of pinene synthase in Escherichia coli. Fuel, 2023, 343: 127872. https://doi.org/10.1016/j.fuel.2023.127872
[27] Wu X, et al. Biosynthesis of pinene in purple non-sulfur photosynthetic bacteria. Microbial Cell Factories, 2021, 20(1): 104. https://doi.org/10.1186/s12934-021-01556-2
[28] Wei LJ, et al. Biosynthesis of alpha-Pinene by Genetically Engineered Yarrowia lipolytica from Low-Cost Renewable Feedstocks. J Agric Food Chem, 2021, 69(1): 275–285. https://doi.org/10.1021/acs.jafc.0c06954
[29] Ma R, et al. Bornyl diphosphate synthase from Cinnamomum burmanni and its application for (+)-borneol biosynthesis in yeast. Frontiers in Bioengineering and Biotechnology, 2021, 9: 631863. https://doi.org/10.3389/fbioe.2021.631863
[30] Ma R, et al. Identification of (-)-bornyl diphosphate synthase from Blumea balsamifera and its application for (-)-borneol biosynthesis in Saccharomyces cerevisiae. Synthetic and Systems

Biotechnology, 2022, 7(1): 490–497. https://doi.org/10.1016/j.synbio.2021.10.004
[31] Ma Y, et al. Targeting pathway expression to subcellular organelles improves astaxanthin synthesis in Yarrowia lipolytica. Metab Eng, 2021, 68: 152–161. https://doi.org/10.1016/j.ymben.2021.05.006
[32] Zhang H, et al. Engineering cellular dephosphorylation boosts (+)-borneol production in yeast. Acta Pharmaceutica Sinica B, 2025, 15(2): 1171–1182. https://doi.org/10.1016/j.apsb.2024.09.011
[33] Tominaga M, et al. Production of borneol, camphor, and bornyl acetate using engineered Saccharomyces cerevisiae. Metabolic Engineering Communications, 2025, 20: e00259. https://doi.org/10.1016/j.meteno.2025.e00259
[34] Cao Y, et al. Biosynthesis and production of sabinene: current state and perspectives. Applied Microbiology and Biotechnology, 2018, 102(4): 1535–1544. https://doi.org/10.1007/s00253-017-8601-7
[35] Wu T, et al. Improvement of sabinene tolerance of Escherichia coli using adaptive laboratory evolution and omics technologies. Biotechnology for Biofuels, 2020, 13(1): 79. https://doi.org/10.1186/s13068-020-01765-9
[36] Jia H, et al. Collaborative subcellular compartmentalization to improve GPP utilization and boost sabinene accumulation in Saccharomyces cerevisiae. Biochemical Engineering Journal, 2020, 164: 107768. https://doi.org/10.1016/j.bej.2020.107768
[37] Dudley QM, et al. In vitro prototyping of limonene biosynthesis using cell-free protein synthesis. Metab Eng, 2020, 61: 251–260. https://doi.org/10.1016/j.ymben.2020.05.004
[38] Zhang R, et al. Synthesis of myrcene using a hybrid cell-free reaction system. Biochemical Engineering Journal, 2024, 208: 109361. https://doi.org/10.1016/j.bej.2024.109361
[39] Korman TP, Opgenorth PH, Bowie JU. A synthetic biochemistry platform for cell free production of monoterpenes from glucose. Nature Communications, 2017, 8(1): 15526. https://doi.org/10.1038/ncomms15526
[40] Gnitka R, Majchrzak W, Wawrzenczyk C. Microbial transformations of (-)-α- and (+)-β-thujone by fungi of Absidia species. Phytochemistry Letters, 2015, 13: 238–248. https://doi.org/10.1016/j.phytolett.2015.05.002
